# Comprehensive Characterization of Secondary Metabolites in Fruits and Leaves of Cloudberry (*Rubus chamaemorus* L.)

**DOI:** 10.3390/metabo13050598

**Published:** 2023-04-27

**Authors:** Anna V. Faleva, Nikolay V. Ul’yanovskii, Aleksandra A. Onuchina, Danil I. Falev, Dmitry S. Kosyakov

**Affiliations:** Laboratory of Natural Product Chemistry and Bioanalytics, Core Facility Center “Arktika”, Northern (Arctic) Federal University, 163002 Arkhangelsk, Russia; a.bezumova@narfu.ru (A.V.F.); a.onuchina@narfu.ru (A.A.O.); d.falev@narfu.ru (D.I.F.)

**Keywords:** *Rubus chamaemorus*, cloudberry, secondary metabolites, extractives, polyphenols, antioxidant activity, non-target screening

## Abstract

Cloudberry (*Rubus chamaemorus* L.) is a circumpolar boreal plant rich in bioactive compounds and is widely used in food and in folk medicine. In this study, a combination of two-dimensional NMR spectroscopy and liquid chromatography–high-resolution mass spectrometry was used for the comprehensive characterization of secondary metabolites in cloudberry lipophilic and hydrophilic extracts. Special attention was paid to the leaf extractives, which are highly enriched in polyphenolic compounds, the content of which reaches 19% in the extract (in gallic acid equivalent). The chemical composition of the polyphenolic fraction is represented mainly by the glycosylated derivatives of flavonoids, hydroxycinnamic (primarily caffeic), gallic (including the structure of galloyl ascorbate) and ellagic acids, catechin, and procyanidins. The contents of aglycones in the polyphenolic fraction were 64 and 100 mg g^−1^ for flavonoids and hydroxycinnamic acids, respectively, while the content of free caffeic acid was 1.2 mg g^−1^. This determines the exceptionally high antioxidant activity of this fraction (750 mg g^−1^ in gallic acid equivalent) and the ability to scavenge superoxide anion radicals, which is 60% higher than that of Trolox. The lower polar fractions consist mainly of glycolipids, which include polyunsaturated linolenic acid (18:3), pentacyclic triterpenic acids, carotenoid lutein, and chlorophyll derivatives, among which pheophytin a dominates. Along with the availability, the high antioxidant and biological activities of cloudberry leaf extracts make them a promising source of food additives, cosmetics, and pharmaceuticals.

## 1. Introduction

Berry plants of the genus *Rubus* (*Rosaceae* family) are widely distributed in nature and are well-known as an important dietary supplement. The highest consumption is typical for raspberry (*R. idaeus*) and blackberry (*R. occidentalis*), cultivated on an industrial scale. In the circumpolar regions of the northern hemisphere, cloudberry (*R. chamaemorus*), which is one of the main components of the vegetation cover of peat bogs, have gained particular popularity [1,2]. In Nordic countries (especially in Norway and Finland) and northern Russia, cloudberry is considered a national delicacy. In addition to the unique spicy flavor, cloudberry possesses a wide range of pharmacological activity and can be used in medicine due to the presence of polyphenolic compounds, among which ellagic acid derivatives (ellagitannins), and phenolcarboxylic acids predominate [3,4]. This distinguishes cloudberries from other berries of the genus, which are characterized by a high content of proanthocyanidins along with ellagitannins [5,6,7]. Cloudberry polyphenols have pronounced anticarcinogenic, antimutagenic, and antioxidant activities [3,8,9], and may also be responsible for their antimicrobial effect, antineoplastic properties, and inhibitory activity against intestinal parasites, as reported in the literature [10,11]. Lashmanova et al. [12] described the geroprotective effects of the cloudberry fruit extract on *Drosophila melanogaster* females, which were associated with the presence of carotenoids in it.

Due to the wide use of cloudberry fruits in the food industry, most of the phytochemical studies have been carried out with them [3,4,13]. At the same time, not only fruits, but also cloudberry leaves demonstrate high biological activity [14]. Given that such plant material can be harvested on a large scale, the chemical composition and bioactive properties of its extractive substances are of considerable interest [5]. Despite this, the secondary metabolites (compounds which are not directly involved in the normal growth and development of the plant) of cloudberry leaves are still extremely poorly studied. The only work which is available in the literature [15] reported the discovery of five polyphenolic compounds in cloudberry leaf ethanolic extracts. Preparative isolation followed by NMR analysis made it possible to identify them as tannins and flavonoid glucuronic glycosides. One compound, 4-O-L-arabinofuranosylellagic acid, was described as a secondary metabolite of cloudberry for the first time.

This study is an attempt to fill this gap and is aimed at a comparative characterization of the aqueous ethanol extracts of cloudberry fruits and leaves, as well as a comprehensive study of the chemical composition and antioxidant activity of leaf secondary metabolites. Its methodological basis is formed by the combination of the most powerful and complementary techniques of two-dimensional NMR spectroscopy and high-performance liquid chromatography–high-resolution mass spectrometry, which has already proven itself in solving problems with regard to non-targeted screening and the identification of extractive substances of moss, compression wood, and birch phloem polyphenols [16,17,18].

## 2. Materials and Methods

### 2.1. Reagents and Materials

Methanol (HPLC grade, Khimmed, Moscow, Russia), methylene chloride (for HPLC, Khimmed, Moscow, Russia), and ethanol (pharm., 96%, BioChemPlant Ltd., Kirov, Russia) were used for the plant biomass extraction and in photochemiluminescence antioxidant activity determination procedures. Acetonitrile (HPLC gradient grade, Khimmed, Moscow, Russia), ACS reagent grade formic acid (≥96%) and orthophosphoric acid (≥85%) purchased from Merck (Darmstadt, Germany), and ultrapure Type I water with a resistivity of 18.2 MΩ·cm obtained in the Milli-Q system (Millipore, Molsheim, France) were used for the preparation of the mobile phase in chromatographic separations and the electrochemical determination of an antioxidant activity. Gallic acid (97.5–102.5%, Sigma-Aldrich, Steinheim, Germany) and Trolox (analytical standard, Analytik Jena AG, Jena, Germany) served as standards in the antioxidant activity and total phenolic content (TPC) determination procedures. The ready-to-use Folin–Ciocalteu reagent solution for the TPC assay was purchased from Sigma-Aldrich (Steinheim, Germany). In NMR studies, deuterated dimethyl sulfoxide (DMSO-*d*_6_, ≥99.8%, Merck, Darmstadt, Germany) and chloroform-*d*_1_ (≥99.8%, Merck, Darmstadt, Germany) were used as sample solvents. Quercetin and caffeic acid (≥95%) purchased from Sigma-Aldrich (Steinheim, Germany) were used as standards in chromatographic semi-quantification of flavonoids and phenolic glycosides. D-Apiose aqueous solution (0.891 M) purchased from Carbosynth Ltd. (Compton, UK) and six monosaccharides (arabinose, galactose, glucose, mannose, xylose, fructose) with a purity of > 98% purchased from Sigma-Aldrich (St. Louis, MO, USA) were used as analytical standards. “Chem. pure” grade sulfuric acid (Komponent-Reaktiv, Moscow, Russia) and barium carbonate (>99.5%, Sigma-Aldrich, St. Louis, MO, USA) were used in the hydrolysis procedure.

### 2.2. Plant Material, Extraction, and Fractionation

The whole plants of cloudberry (*R. chamaemorus*) were collected in the «Ilasskoye» raised bog (64.3° N, 40.6° E) in the Primorsky district of the Arkhangelsk region, Russia in July 2022 (Supplementary material, Figure S1). The identification of the botanical material was carried out according to the herbarium of the Northern (Arctic) Federal University. Fruits and leaves were separated and frozen immediately after delivery to the laboratory. The moisture content determined on an HG53 halogen moisture analyzer (Mettler-Toledo, Columbus, OH, USA) was 82% and 65% for the fruits and leaves, respectively. The portion of leaves was freeze-dried for the subsequent extraction with methanol–dichloromethane mixture.

Secondary metabolites were extracted from the finely ground and carefully averaged plant material (10 g dry weight) with extractant/sample ratio of 10 mL g^−1^ in three 20 min stages under sonication (35 kHz) in ultrasonic bath (Sapphire, Moscow, Russia) with 70% aqueous ethanol and methanol–dichloromethane mixture (1:1, *v*/*v*). The latter extractant was chosen for the subsequent polarity-based fractionation of the leaf extractive substances [19]. The obtained water–ethanol extracts of leaves and fruits were evaporated under vacuum on a rotary evaporator to a volume of 5–7 mL, then mixed with an excess of water (100–150 mL), frozen with liquid nitrogen, and then lyophilized in a FreeZone Triad freeze dryer (Labconco, Kansas City, MO, USA). The obtained MeOH/DCM extract of leaves (~100 mL) was mixed with 1 g of octadecyl silica Polygoprep 60–50 C_18_ with particle size 40–63 μm (Macherey-Nagel, Duren, Germany) and evaporated under vacuum to dryness. The resulting dry mixture was transferred in a glass chromatography column (200 × 20 mm) filled with the same adsorbent (bed weight 5 g). Then, the adsorbed analytes were sequentially eluted with four solvents (50 mL each) in order of decreasing polarity water, water–methanol mixture (1:1, *v*/*v*), methanol, and methanol–dichloromethane mixture (1:1, *v*/*v*) to obtain the fractions F1, F2, F3, and F4, respectively. Each fraction was concentrated under vacuum on a rotary evaporator to a volume of several milliliters and diluted with an excess of water (except F1). The obtained suspensions were immediately frozen and lyophilized in the same way as water–ethanol extracts. After weighing, the dry extracts were stored in amber glass vials at 4 °C.

### 2.3. Monosaccharide Analysis

Seven target monosaccharides (glucose, xylose, galactose, arabinose, mannose, fructose, and apiose) were determined by high-performance ligand exchange chromatography (HPLEC) with refractometric detection according to the procedure described earlier [20]. A Nexera XR HPLC system (Shimadzu, Kyoto, Japan) which consisted of a DGU-5A vacuum degasser, an LC-20AD chromatographic pump, a SIL-20AC autosampler, a CTO-20AC column thermostat, and an RID-20A refractometric detector was used. The chromatographic separation was carried out at 75 °C on a Rezex RPM-Monosaccharide Pb^+2^ column (Phenomenex, Torrance, CA, USA), 300 × 7.8 mm, using the pure water (flow rate 0.6 mL min^−1^) as a mobile phase. The injection volume was 10 µL. The system control and quantification of the analytes were performed using a LabSolution software (Shimadzu, Kyoto, Japan). The HPLC system was calibrated using the aqueous standard solutions of the monosaccharides mixture with concentrations of 10–1000 mg L^−1^.

Free monosaccharides determination was carried out by direct injection of the centrifuged aqueous solutions of the obtained dry extracts. The total monosaccharides content was determined after a preliminary two-stage acid hydrolysis of the extracts according to the following procedure. The dry extract sample (10 mg) was placed in a 4 mL conical glass vial, poured with 100 µL of 72% sulfuric acid, and kept at 30 °C for 60 min in a Reacti-Therm reaction system (Thermo Scientific, Waltham, MA, USA) equipped with a heating block and a magnetic stirring module. Then, 2.5 mL of water was added and the reaction mixture was heated to 100 °C, kept for 3 h under continuous stirring, and allowed to cool down at ambient conditions. After neutralizing the acid by adding an excess of BaCO_3_ and centrifugation, the obtained solution was injected to the HPLC system. All assays were performed in triplicate. 

### 2.4. Spectrophotometric Determination of Total Phenolic Content and Pigments

The total phenolic content (TPC) was determined by Folin–Ciocalteu assay [21] with minor modifications. The methanolic solution (1 mL) of the dry sample with the concentration of 0.1–0.2 mg mL^−1^ was mixed with 5 mL of Folin–Ciocalteu reagent (10% aqueous solution) and kept for 5 min at room temperature. Then, 4 mL of sodium bicarbonate solution (60 g L^−1^) was added and the mixture was allowed to stand for 60 min. The absorbance of the obtained reaction products was measured at the wavelength of 750 nm on a Specord 250 Plus double-beam UV-Vis spectrophotometer (Analytik Jena AG, Jena, Germany) in a quartz cell with 1 cm optical path. The calibration curve was constructed using the gallic acid as a standard. All assays were performed in triplicate.

### 2.5. Antioxidant Activity Determination

An amperometric determination of the antioxidant activity (AOA) was carried out on a Blizar AOA analyzer (Interlab, Moscow, Russia) consisting of a chromatographic pump, a loop injector, and an electrochemical detector with a glassy carbon working electrode [22]. The sample was dissolved in 50% aqueous acetonitrile containing 2.2 mM ortho-phosphoric acid. The same solvent was used as a mobile phase into which the sample solution (100 μL, 1 mg L^−1^) was injected at a flow rate of 1.2 mL min^−1^. The detection was carried out in a direct current mode with a working electrode potential of 1.3 V. Standard solutions of gallic acid (0.2–4 mg L^−1^) were used for the calibration curve construction. All measurements were performed in at least five replicates. 

The superoxide anion radical scavenging activity was measured using the sample solution in methanol (1 mg L^−1^) by the photochemiluminescence (PCL) method on a Photochem analyzer (Analytik Jena AG, Jena, Germany) according to the known procedure [23]. The dedicated ACL reagent kit (Analytik Jena AG, Jena, Germany) was used. The calibration was performed immediately before the analysis using the Trolox standard solutions in methanol (0.5–3 mg L^−1^). Antioxidant activity PCL assays were performed in triplicate.

### 2.6. Two-Dimensional NMR Spectroscopy

The dry sample of the extract/fraction (30 mg) was dissolved in 550 μL of DMSO-*d*_6_ (CDCl-*d*_1_ in the case of the fraction F4) and placed in the 5 mm (i.d.) NMR tube. HSQC (heteronuclear single quantum correlation) and HMBC (heteronuclear multiple bond correlation) two-dimensional ^1^H-^13^C NMR spectra were recorded at 298 K on an AVANCE III 600 spectrometer (Bruker, Ettlingen, Germany) with an operating frequency for protons of 600 MHz. Bruker Topspin 3.2 software (Bruker, Ettlingen, Germany) was used for the registration and for processing the experimental data. The specific experimental and processing parameters are presented in previous work [17]. The cross-peak assignment to identify the specific structures was performed by combining data from the HSQC and HMBC spectra using an ACD/Structure Elucidator expert system software (ACD/Labs, Toronto, ON, Canada), including NMR spectral database.

### 2.7. High-Performance Liquid Chromatography–High-Resolution Mass Spectrometry

The screening for low-molecular-weight metabolites in F2–F4 fractions was performed by high-performance liquid chromatography–high-resolution mass spectrometry (HPLC–HRMS) on an HPLC–HRMS system consisting of an LC-30 (Shimadzu, Kyoto, Japan) liquid chromatograph with UV-Vis diode array spectrophotometric detector and an Orbitrap ID-X high-resolution mass spectrometer (Thermo Scientific, Waltham, MA, USA) with linear and orbital ion trap mass analyzers and an OptaMax NG ion source equipped with a heated electrospray (HESI) probe.

The chromatographic separation was carried out on a Nucleodur PFP column (Macherey-Nagel, Duren, Germany), 150 × 2 mm, 1.8 µm particle size, with a pentafluorophenyl reversed stationary phase. The mobile phase was a mixture of water (A) and acetonitrile (B), both containing 0.1% formic acid. The flow rate was 0.3 mL min^−1^, column temperature −40 °C. In the analysis of F2 and F3 fractions, the following gradient elution program was used: 0–3 min, 10% B; 3–40 min, linear ramp to 100% B; 40–45 min, 100% B. In the case of fraction F4 containing least polar analytes, the gradient elution program was modified as follows: 0–3 min 40% B; 3–25 min linear ramp to 100% B; 25–30 min 100% B. An injection volume was 2.0 µL. A wavelength range of 220–800 nm, a spectral resolution of 4 nm, and an acquisition rate of 10 Hz were used in the spectrophotometric detection.

High-resolution mass spectrometry detection was carried out in positive (ESI+) and negative (ESI−) ion electrospray ionization modes. The following ion source parameters were applied: spray voltage, 3.5 (ESI+) and 2.5 (ESI−) kV; sheath, auxiliary, and sweep gas (N_2_) flow rates, 50, 10, and 2 arb. units, respectively; ion transfer tube and vaporizer temperature, 325 and 350 °C, respectively; S-lens RF level, 60%. Mass spectra were recorded in the *m*/*z* range of 100–1000 using an orbital ion trap; the mass analyzer resolving power was set to 120,000 (at *m*/*z* 200). Tandem (MS/MS) mass spectra were recorded in the data-dependent acquisition mode. The ions whose signal intensities exceeded a threshold value of 1.0 × 10^5^ cps were selected as precursor ions for MS/MS experiments. Higher-energy collision-induced dissociation (HCD) in the stepped collision energy mode (20, 35, and 60%) with the mass analyzer resolving power of 30,000 were used in the tandem mass spectrometry analysis. To achieve the highest mass accuracy, an internal mass scale calibration (Easy-IC mode with a fluoranthene as a standard) was used. The instrument control and data acquisition were performed using Xcalibur 4.4 software (Thermo Scientific, Waltham, MA, USA). Non-target screening and the identification of the detected compounds were carried out using Compound Discovery 3.3 software package (Thermo Scientific, Waltham, MA, USA) with an online search in the Chemspider, PubChem, Lotus, mzCloud, and Lipid Maps databases. The following constraints were applied in the accurate mass-based elemental composition determination procedure: the maximum allowed deviation of the measured *m*/*z* from the theoretical value is 3 ppm; the maximum numbers of C, H, O, N, S, and P atoms are 100, 200, 50, 10, 2, and 2, respectively.

The samples were prepared immediately before the analysis. One milligram of the extract was dissolved in 1 mL of methanol, the solution was centrifuged for 5 min at 14,000× *g* rpm, and the supernatant was injected into the HPLC–HRMS system.

## 3. Results and Discussion

### 3.1. Comparative Analysis of Fruit and Leaf Ethanolic Extracts

At the initial stage of the work, an ultrasonic extraction of the plant material with 70% aqueous ethanol was used to isolate the widest range of extractive substances from cloudberry fruits and leaves for the purposes of their general characterization and comparison. The choice of the extractant was based on the literature data [24,25,26,27] demonstrating the high efficiency of this solvent in the extraction of secondary metabolites that differ greatly in polarity, including in pharmaceutical applications. Surprisingly, despite the obvious differences between the two types of plant tissues, they showed almost identical yields of extractives, which amounted to 55 ± 4%. At the same time, the chemical composition and antioxidant properties of the two obtained extracts (Table 1) differed very significantly.

As expected, the most important components of the obtained extracts were carbohydrates, which, in the form of free monosaccharides, accounted for ~26 and ~10% of the total mass of extractive substances in fruits and leaves, respectively. Both samples were characterized by a sharp dominance (80% of free monosaccharides) of glucose. In the case of fruits, the remaining sugars were mainly fructose, while the contents of xylose, galactose, and arabinose were comparable to those found in the leaf extract. To assess the presence of bound forms of monosaccharides, the obtained extracts were reanalyzed after the two-stage acid hydrolysis. For the fruits, no significant differences were observed before and after hydrolysis, with the exception of arabinose, the content of which increased by an order of magnitude (19 ± 2 mg g^−1^). In the case of leaves, a completely different pattern was observed: after hydrolysis, the total content of monosaccharides increased two-fold (for the galactose—by an order of magnitude), which indicates the presence of significant amounts of oligosaccharides or glycosylated secondary metabolites in the extract.

Evidently, the most significant difference between the extracts is in the content of the polyphenolic compounds (TPC) determined by the Folin–Ciocalteu method. Thus, TPC in leaf extracts turned out to be 26 times higher than in fruits and reached almost 20% of the dry extract weight. The presence of large amounts of polyphenols caused the observed high antioxidant activity of the leaf extract. Its values determined by two independent methods (electrochemical and PCL assays characterizing the ability to the single electron transfer and superoxide anion radical quenching by the hydrogen atom transfer, respectively) were identical within the measurement error and close to the TPC. A completely different pattern was observed for the fruits: the antioxidant activity was more than an order of magnitude higher than the TPC value. This can be explained by the fact that in the case of fruits, the main contribution to the antioxidant activity of the extract is made not only by polyphenols, but also by metabolites belonging to other classes. Among them, the ascorbic acid, the content of which in cloudberries is high [28], should be noted.

**Figure 1:**
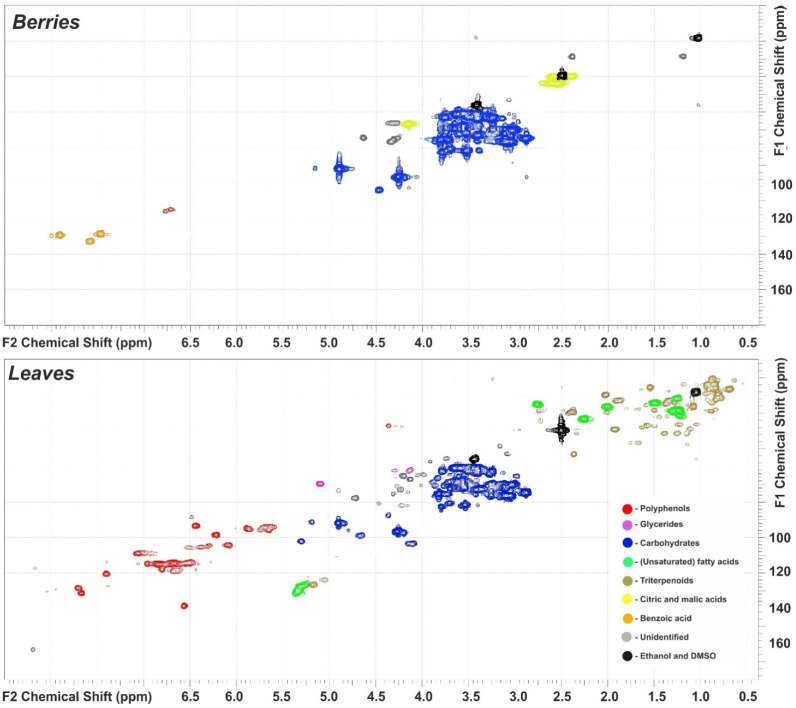
2D HSQC spectra of cloudberry fruit (**top**) and leaf (**bottom**) ethanol–water extracts.

The components presented in Table 1 make up no more than half of the total mass of extractive substances of both fruits and leaves. The rest is represented by minerals (ash), lipids, pigments, and some other compounds. The overall picture of the chemical composition of the obtained extracts is well reflected in their two-dimensional (HSQC) ^1^H–^13^C NMR spectra (Figure 1) evincing clear differences in major classes of the secondary metabolites. Thus, in the spectrum of the fruit extract, the intense signals of sugars (primarily, glucose and fructose) at δC/δH 50–105/2.5–5.0 predominate, which correlates with the results obtained by the HPLEC assay. In the aromatic region (δC/δH 100–140/6.0–8.0 ppm), the three intense cross-peaks were attributed to benzoic acid, which had previously been identified in cloudberry extracts [4]. The much weaker signal at δC/δH 115/6.7 ppm belongs to polyphenolic structures. The distinctive features of the leaf extract 2D NMR spectrum are the appearance of the presence of a large number of cross-peaks in the aromatic region, and an emergence of the signal-saturated area in the aliphatic region (δC/δH 8.0–56/0.5–3.0 ppm) of the spectrum. The signals in the first area indicate the presence of various polyphenolic compounds, including proanthocyanidins and flavonoids. The second area contains cross-peaks mainly related to lipids and, presumably, triterpenoids.

This suggests that from the point of view of the search for compounds with high biological activity and antioxidant properties, cloudberry leaves, which are enriched in polyphenolic compounds, are of great interest. In this regard, further studies focused on a detailed characterization of their chemical composition by 2D NMR and HPLC–HRMS techniques.

### 3.2. Polarity-Based Fractionation of Leaf Extract and General Characterization of Fractions

In order to reduce the content of carbohydrates in the extract and focus on the most promising (in terms of biological activity) polyphenolic and less polar compounds, cloudberry leaves were subjected to ultrasonic extraction with a mixture of methanol and dichloromethane (1:1). Subsequent column chromatography fractionation of the extract by sorption on an octadecyl silica and successive elution with water, water–methanol mixture (1:1), neat methanol, and methanol–dichloromethane mixture (1:1) made it possible to obtain four fractions (F1–F4, respectively), differing in the polarity with the total yield of 19.5% (Table 2).

As expected, fraction F1 was a mixture of monosaccharides and was not of significant interest. According to the HPLEC analysis, it consisted of glucose (274 mg g^−1^), xylose (44 mg g^−1^), galactose (37 mg g^−1^), arabinose (14 mg g^−1^), and fructose (73 mg g^−1^). Apiose was also detected in trace amounts (0.46 mg kg^−1^), which indicates the likely presence of apiosides. It did not contain polyphenolic compounds; thus, the antioxidant activity was negligible.

The most attention should be drawn to the fraction F2, the yield of which exceeded 6% of the oven-dry plant material. More than half of the mass of this fraction was made up of polyphenolic compounds, which provide exceptionally high antioxidant activity. Particularly noteworthy is the exceptional ability of F2 to the superoxide anion radical scavenging (PCL assay results), which exceeded this parameter for Trolox by 60%.

The less polar fraction F3 also showed a noticeable antioxidant activity, which was, however, 5–8 times less, compared to F2. This is due to the presence of polyphenols (10.7%) in its composition, as well as a certain amount of carotenoids. The least polar components make up the fraction F4, which did not possess significant antioxidant activity (amperometric assay) and, apparently, contained mainly lipids. Chlorophyll derivatives and some carotenoids were also concentrated in it.

### 3.3. Polyphenolic Compounds in the Fraction F2

**Figure 2:**
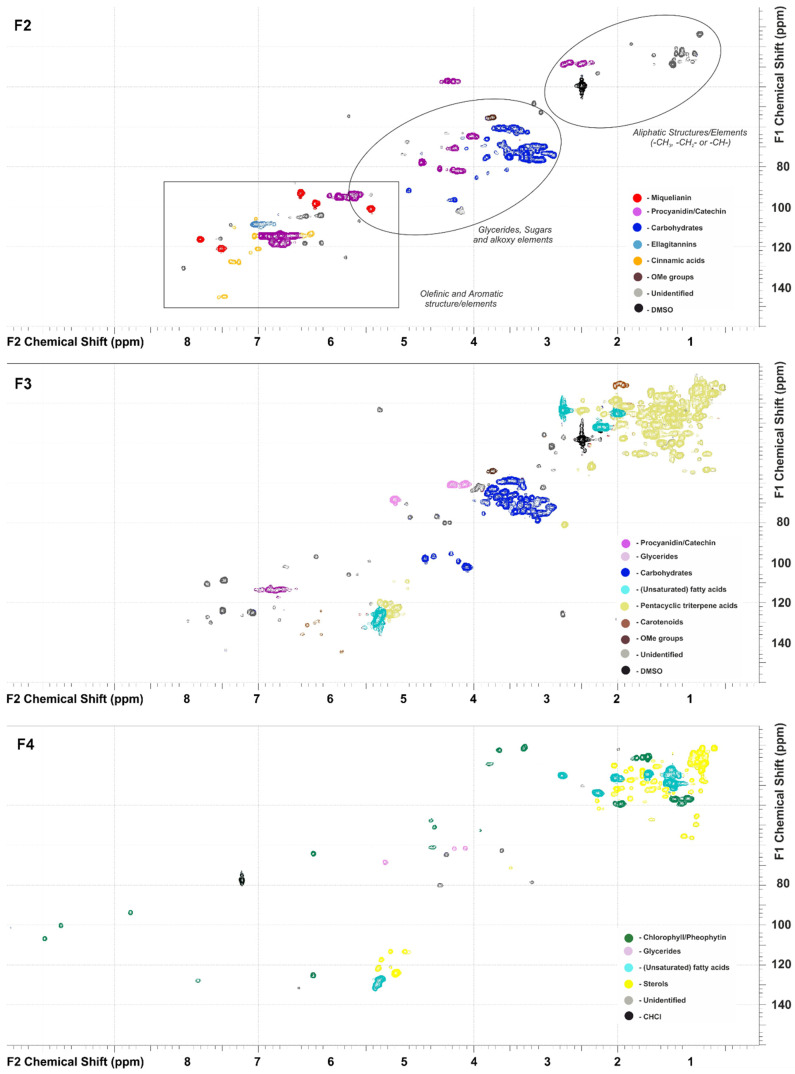
2D HSQC NMR spectra of the cloudberry leaf extract fractions F2–F4.

The HSQC NMR spectrum of the fraction F2 of the cloudberry leaf extract (Figure 2) contains characteristic sets of cross-peaks related to several major groups of secondary phenolic metabolites. These include flavonoids, procyanidins, ellagitannins, cinnamic acids, and sugar moieties. The further processing of HSQC and HMBC data involving an electronic expert system and NMR databases made it possible to identify some major compounds or structural fragments in more complex substances. In particular, intense signals at δC/δH 116.5/7.81, 121.2/7.52, 93.4/6.41, 98.6/6.21, and 101.4/5.43 indicate the presence of the flavonol glucuronide miquelianin as the main constituent of the fraction F2, which is consistent with the literature data [15]. Catechin and the structures of four hydroxycinnamic acids (*p*-hydroxycinnamic, ferulic, sinapic, and caffeic) in the corresponding glycosides have also been reliably identified. Despite the unambiguous detection of the procyanidin structure, NMR cannot provide an information on the degree of polymerization of the corresponding oligomers. It should be noted that the quantitative assessment of the obtained NMR spectra showed the predominance of flavonoids and procyanidins, while ellagitannins dominate among the polyphenols of cloudberry fruits [29].

**Figure 3:**
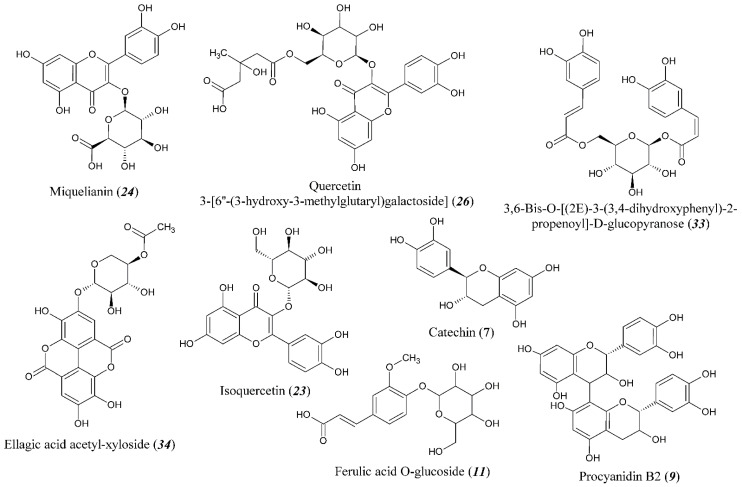
Structural formulas of some polyphenolic compounds identified in the fraction F2 (the number in parentheses corresponds to the compound number in Table 3).

The detailed molecular level characterization of the F2 chemical composition by HPLC–HRMS (Figure S2) allowed for the detection and tentative identification of 38 phenolic compounds with a retention time (t_R_) from 3 to 18 min (Table 3). The structural formulas of some of them are shown in Figure 3. The most intense signal, exceeding the peaks of other analytes by an order of magnitude, was observed for compound **24** (hereinafter in the section, the numbers of chemical compounds correspond to Table 3) with a retention time of 10.2 min and the elemental composition C_21_H_18_O_13_.

On the basis of the tandem mass spectrum containing the signals of [C_15_H_9_O_7_]^−^ and [C_6_H_7_O_6_]^−^ product ions related to quercetin and glucuronic acid residue, respectively, it was unambiguously identified as the aforementioned miquelianin (quercetin 3-O-glucuronide). Another major component of the extract with the elemental composition C_21_H_18_O_12_ (**29**) was also assigned to glucuronides; however, the flavonoid kaempferol acts as an aglycone in its structure. In addition to these two compounds, the fraction F2 contained a wide range of flavonoids, the vast majority of which were in the form of glycosides, mainly hexosides. In particular, the compound C_21_H_20_O_12_ with t_R_ = 10.1 min (**23**) was identified as isoquercetin (isoquercitrin) since in its MS/MS spectrum, the signals of quercetin and hexose monosaccharide were observed. It should be noted that the sugar residue in glycosylated flavonoids can be modified by dicarboxylic acids—glutaric and malonic. Representatives of such structures are compounds **26** and **27**, identified as quercetin 3-[6′-(3-hydroxy-3-methylglutaryl)galactoside] and quercetin 3-(6′-malonylgalactoside), respectively. In some cases, flavonoids and other phenolic compounds were modified with gallic and ascorbic acids (galloyl ascorbates). In this regard, the corresponding derivatives of dihydromyricetin (**28**) and taxifolin (**36**) can be cited as examples.

Unlike flavonoids with a flavone backbone, compounds with an isoflavane structure (catechins) are present in a free (non-glycosylated) form, resulting in intense signals on the chromatogram. These include catechin itself (**7**) and its spatial isomer epicatechin (**12**), as well as a number of dimeric and trimeric structures of procyanidins B and C (**4, 9, 10, 18**). Procyanidin B2 and epicatechin are the most abundant compounds of isoflavane group and contribute to the second and third peaks in intensity on the chromatogram, respectively. Of the catechin derivatives, epicatechin galloyl ascorbate (**3**) should be noted since such compounds can make a significant contribution to the antioxidant activity of cloudberry extracts.

Gallic acid plays an important role in the formation of cloudberry secondary phenolic metabolites. Although it was not found in a free form, in addition to the gallic acid flavonoid derivatives noted above, its dimerization product, ellagic acid (**22**), was identified, including in the form of glycosides **21** and **34**. Gallic acid is also responsible for the formation of the most complex detected compound, presenting a signal of a doubly charged [M − 2H]^2–^ ion with *m*/*z* 934.0712 in the mass spectrum, which corresponds to the parent compound with the molecular weight of 1871 Da and the elemental composition C_82_H_54_O_2_. Considering that its tandem mass spectrum demonstrates the sequential loss of gallic acid and hexose monosaccharide along with the formation of gallic acid dimers, this compound was identified as a representative of ellagitannins, namely Sanguiin H-6, previously found in cloudberry fruits [3].

A large group of detected phenolic compounds, consisting of ten representatives, are glycosylated derivatives of hydroxycinnamic acids—ferulic, sinapic, and especially caffeic, since the latter account for more than 75% of the total peak area of compounds of this class in the chromatogram. Both hexoses and uronic acids can act as a sugar moiety, which can be illustrated by compounds **1** and **2** assigned to caffeic acid glucuronide and glucoside, respectively. Caffeic acid is also present in a free form in small amounts (**13**).

Since the UV absorption spectra of phenolic compounds and their glycosylated derivatives in the region of 250–400 nm differ slightly while flavonoids and hydroxycinnamic acids predominantly absorb the radiation at different wavelengths (λ_max_ = 370 and 320 nm, respectively), it is possible to semi-quantitatively determine the total contents of the corresponding aglycones. For this purpose, HPLC–UV chromatograms recorded for the indicated wavelengths were used to determine the total peak areas for both flavonoid and hydroxycinnamic acid derivatives, and an HPLC system was calibrated using external standards of quercetin and caffeic acid. The obtained values for the contents of the corresponding aglycones were 64 and 100 mg g^−1^, respectively, while the content of free caffeic acid was 1.2 mg g^−1^.

### 3.4. Less Polar Compounds in the Fractions F3 and F4

Cross-peaks observed in the 2D NMR spectra of the fraction F3 indicate that it predominantly consists of unsaturated fatty acid (glycoside) glycerides. The high density of signals in the aliphatic region of the HSQC NMR (Figure 2) spectrum also suggests the presence of various triterpene derivatives. In the aromatic region, weak signals from polyphenols and double bonds, presumably related to carotenoids, were detected. Based on the data presented in the F4 spectrum, it is expected that this fraction is dominated by unsaturated fatty acid glycerides, steroids, and, most of all, chlorophyll derivatives. The last of which cause the intense characteristic cross-peaks at δC/δH 10.95/3.34; 12.04/3.69; 64.06/6.28; 93.34/8.81; 99.88/9.78; and 106.40/10.01 ppm.

The HPLC–HRMS chromatograms obtained in both positive and negative ion modes confirmed the data of NMR spectroscopy and allowed for the identification of 48 major lipophilic compounds belonging to the various classes: fatty acid glycerides, terpenoids, steroids, carotenoids, and chlorophyll derivatives (Figure 2, Table 4). The fraction F3 was the richest in its chemical composition and contained 47 tentatively identified compounds. The more polar ones (**1**–**4**, **6**, **7**; hereinafter in the section, the numbers of chemical compounds correspond to Table 4) were eluted first in the chromatogram and also found in the F2 fraction (Table 3). It should be noted that while the peak areas of flavonoids in the F3 chromatogram were significantly less than those for the fraction F2, the opposite picture was observed for ellagic acid and its acetyl xyloside; the main part of this substance was concentrated in the fraction F3, providing its high antioxidant activity. Another polyphenolic component of the fraction F3 is compound **9** with the elemental composition C_30_H_25_O_13_, which results in fragments of hydroxycinnamic acid, kaempferol, and hexose monosaccharide in the tandem mass spectrum, and is presumably identified as tiliroside, a glycosyloxyflavone that is kaempferol attached to *p*-hydroxycinnamoyl glucoside.

The largest proportion of the total peak area on the F3 chromatogram belongs to lipids of various classes, characterized by retention times in the range of 20–32 min (Figure S2). Among them, galactolipids, namely digalactosylmonoacylglycerols (DGMG), monogalactosylmonoacylglycerols (MGMG), monogalactosyldiacylglycerols (MGDG), and digalactosyldiacylglycerols (DGDG), including positional isomers, predominate as important cell membrane constituents. A specific feature of the detected galactolipids noted earlier [30] is the formation of the ammonium cationized molecules ([M + NH_4_]^+^) instead of protonated ones in ESI(+), even in the absence of ammonium salts in the mobile phase. Fatty acids in their composition are represented mainly by linolenic acid (18:3) as well as residues of linoleic (18:2), and, in some cases, stearic (18:0) and palmitic (16:0) acids. Linolenic acid is also a part of the detected uronic acid-based glycolipid GlcADG (**42**) and a compound **45** belonging to the glycerophospholipid class PG (18:3/16:1). The latter, due to the presence of orthophosphoric acid residue in its structure, is detected only in the ESI(–) mode. The predominance of polyunsaturated ω-3 linolenic acid possessing anti-carcinogenic, lipid metabolism regulation, anti-inflammatory, anti-obese, and antioxidant activities [31] significantly increases the value of the lipophilic fraction of cloudberry leaf extract and makes it promising for various pharmaceutical and dietary applications.

Another large group of secondary metabolites in the F3 fraction is represented by pentacyclic triterpenoids, which result in a group of intense peaks in the chromatogram in the retention time range of 15–20 min. Among them, four compounds with the elemental composition C_30_H_48_O_6_ predominate, which are triterpenic acids, possessing in their structure, along with the carboxylic group, four hydroxyls (the loss of CO_2_ and four water molecules in the tandem mass spectrum with further fragmentation of the hydrocarbon residue was observed). Due to the possibility of the existence of a large number of positional isomers and the nonspecificity of carbon skeleton fragmentation, the reliable identification of such compounds is difficult. Based on the available literature data on the presence of the *Rubus* family in the tissues of plants and differences in polarity (the order of retention on the reversed stationary phase), we identified them as hydroxyasiatic (**11**), tomentosic (**12**), myrianthic (**13**), and hydroxyeuscaphic (**14**) acids. In addition to triterpenoids, two steroids were also found, giving an intense peak at the end of the chromatogram with a retention time of 33.4 min. They were annotated as hydroxystigmastanones (**43, 44**).

Pigments in the fraction F3 are mainly represented by pheophorbide a (**31**) and its methylated derivative (**40**), as well as carotenoids lutein (**34**) and anhydrolutein (**33**). More polar carotenoid erythroxanthin (**22**) was also found in small amounts. As impurities, these compounds (with the exception of erythroxanthin) are also present in the fraction F4. However, the basis of its chemical composition is pheophytins *a* and *b*, which account for about 90 and 8% of the total intensity of the peaks in the chromatogram, respectively (Figure S2). Half of the remaining 2% is hydroxypheophytin A.

## 4. Conclusions

The fruits and leaves of cloudberry (*Rubus chamaemorus*) contain more than 50% of extractives recovered with aqueous ethanol, while the chemical compositions of the extracts obtained from them are very different. Compared with fruits, the leaves are highly enriched in polyphenolic compounds, the content of which in the extract reaches 19% (in gallic acid equivalent). According to 2D NMR and HPLC–HRMS analyses, the polyphenolic fraction of leaf extractives is represented mainly by glycosylated derivatives of flavonoids, hydroxycinnamic (primarily caffeic), gallic (including the structure of galloyl ascorbate) and ellagic acids, catechin, and procyanidins. This determines the exceptionally high antioxidant activity of this fraction and the ability to scavenge superoxide anion radicals, which is 60% higher than that of Trolox. The less polar fractions consist mainly of glycolipids, which include polyunsaturated linolenic acid (18:3), pentacyclic triterpenic acids, carotenoid lutein, and chlorophyll derivatives, among which pheophytin *a* dominates.

Along with the availability, the high antioxidant and biological activities of the extractive substances of cloudberry leaves make them a promising raw material for the production of food additives, cosmetics, and pharmaceuticals. Further research in this direction should be focused on the development of methods for the most complete and selective isolation of various groups of extractive substances from plant material, as well as a detailed description of the biological activity of the obtained extracts.

## Figures and Tables

**Table 1:** Chemical composition and antioxidant activity of cloudberry fruits and leaves’ water–ethanol extracts (mg g^−1^).

Parameter/Compound	Fruits	Leaves
TPC	7.0 ± 1.3	187 ± 4
Antioxidant Activity		
Electrochemical assay (in gallic acid equivalent)	85 ± 6	230 ± 20
Photochemiluminescence assay (in Trolox equivalent)	88 ± 2	250 ± 60
Free Monosaccharides		
Glucose	198 ± 15	76 ± 8
Xylose	8.4 ± 1.3	9.6 ± 0.8
Galactose	4.2 ± 0.5	2.8 ± 0.3
Arabinose	2.1 ± 0.3	3.8 ± 0.4
Fructose	47 ± 6	8.8 ± 1.1

**Table 2:** Fractions of the cloudberry leaf dichloromethane–methanol extract and their general characteristics.

Parameter	F1	F2	F3	F4
Yield, %	8.5	6.4	2.9	1.6
TPC, mg g^−1^	-	570 ± 50	107 ± 6	-
Total carotenoids, mg g^−1^	-	-	3.1 ± 0.1	0.93 ± 0.03
Pheophytin (A + B), mg g^−1^	-	-	1.71 ± 0.05	3.5 ± 0.2
Antioxidant Activity				
Amperometric assay, mg g^−1^	-	750 ± 20	157 ± 9	-
PCL assay, mg g^−1^	-	1590 ± 20	201 ± 1	-

**Table 3:** Major compounds in the fraction F2 and their tentative identification by HPLC–ESI(−)–HRMS.

No	t_R_, min	Formula	*m*/*z*	Δ*m*/*z*, ppm	Peak Area, ×10^7^	Assumed Compound
1	3.0	C_15_H_18_O_9_	341.0874	−1.09	5.45	Caffeic acid 4-O-glucuronide
2	3.2	C_15_H_18_O_9_	341.0875	−1.00	5.26	Caffeic acid 3-glucoside
3	3.5	C_21_H_20_O_12_	463.0882	−0.01	4.90	Epigallocatechin ether of ascorbic acid
4	3.6	C_30_H_26_O_12_	577.1350	−0.24	2.99	Procyanidin B-type (isomer)
5	3.6	C_15_H_18_O_9_	341.0874	−1.09	5.41	1-Caffeoyl-β-D-glucose
6	3.8	C_45_H_38_O_18_	865.1983	−0.27	1.02	Procyanidin C-type (isomer 1)
7	4.1	C_15_H_14_O_6_	289.0715	−0.79	16.9	Catechin
8	4.3	C_20_H_28_O_12_	459.1509	0.31	0.94	Caffeic acid glycosylated with 2,3,4-Trihydroxy-2-methylbutyl β-D-gulopyranoside
9	4.4	C_30_H_26_O_12_	577.1345	−1.19	43.1	Procyanidin B2
10	5.0	C_45_H_38_O_18_	865.1980	−0.62	3.62	Procyanidin C-type (isomer 2)
11	5.1	C_16_H_20_O_9_	355.1034	−0.43	1.12	Ferulic acid O-glucoside
12	5.2	C_15_H_14_O_6_	289.0714	−1.32	36.7	Epicatechin
13	5.2	C_9_H_8_O_4_	179.0349	−0.39	0.69	Caffeic acid
14	5.7	C_16_H_20_O_9_	355.1033	−0.35	1.05	Ferulic acid derivative I *^,1^
15	6.3	C_17_H_22_O_10_	385.1140	−0.1	1.35	Sinapoyl D-glucoside
16	6.6	C_16_H_20_O_9_	355.1035	0.0	0.99	Ferulic acid derivative II *^,1^
17	7.0	C_17_H_22_O_10_	385.1139	−0.25	1.24	Sinapic acid derivative I *^,2^
18	7.2	C_45_H_38_O_18_	865.1982	−0.41	1.32	Procyanidin C-type (isomer 3)
19	7.7	C_17_H_22_O_10_	385.1140	0.0	1.05	Sinapic acid derivative II *^,2^
20	8.4	C_82_H_54_O_52_	934.0712 **	−0.39	15.5	Sanguiin H-6 II
21	9.6	C_19_H_14_O_12_	433.0410	−0.68	17.4	Arabinofuranosylellagic acid
22	9.9	C_14_H_6_O_8_	300.9989	−0.3	6.88	Ellagic acid
23	10.1	C_21_H_20_O_12_	463.0884	0.35	8.57	Isoquercetin
24	10.2	C_21_H_18_O_13_	477.0668	−1.34	162	Miquelianin
25	10.2	C_21_H_16_O_13_	475.0524	−1.19	3.60	2-[2-[2-(3,4-Dihydroxyphenyl)-5,7-dihydroxy-4-oxochromen-3-yl]oxy-2-oxoethyl]-2-hydroxybutanedioic acid
26	10.8	C_27_H_28_O_16_	607.1304	−0.14	4.26	Quercetin 3-[6′-(3-hydroxy-3-methylglutaryl) galactoside]
27	10.8	C_24_H_22_O_15_	549.0886	0.05	7.18	Quercetin 3-(6′-malonylgalactoside)
28	10.9	C_28_H_22_O_17_	629.0782	−0.38	4.31	Dihydromyricetin ether of galloyl ascorbate
29	11.0	C_21_H_18_O_12_	461.0722	−0.68	14.5	Kaempferol 3-glucuronide
30	11.1	C_28_H_36_O_11_	547.2188	0.60	0.37	(1S,2R)-2-Hydroxy-3,5,5-trimethyl-4-[(1E)-3-oxo-1-buten-1-yl]-3-cyclohexen-1-yl 6-O-[(2E)-3-(3,4-dihydroxyphenyl)-2-propenoyl]-β-D-glucopyranoside
31	11.2	C_30_H_28_O_15_	627.1354	−0.27	0.64	Taxifolin ether of leucodrin
32	11.3	C_24_H_24_O_11_	487.1245	−0.28	4.97	Beta-D-glucopyranose 1-(4-trans-coumaric acid)-2-(3,4-dihydroxy-trans-cinnamate)
33	11.4	C_24_H_24_O_12_	503.1196	0.26	3.27	3,6-Bis-O-[(2E)-3-(3,4-dihydroxyphenyl)-2-propenoyl]-D-glucopyranose
34	11.5	C_21_H_16_O_13_	475.0519	0.27	2.91	Ellagic acid acetyl-xyloside
35	11.6	C_28_H_36_O_11_	547.2189	0.93	0.77	Isomer of **30**
36	11.8	C_28_H_22_O_16_	613.0835	−0.02	0.49	Taxifolin ether of galloyl ascorbate
37	12.1	C_28_H_36_O_11_	547.2188	0.60	0.79	(2R,3E)-4-[(1S)-1-Hydroxy-2,6,6-trimethyl-4-oxo-2-cyclohexen-1-yl]-3-buten-2-yl 6-O-[(2E)-3-(3,4-dihydroxyphenyl)-2-propenoyl]-β-D-glucopyranoside
38	13.6	C_23_H_24_O_13_	507.1144	−0.01	1.66	5,8-Dihydroxy-4-oxo-1,2,3,4-tetrahydro-1-naphthalenyl 6-O-(3,4,5-trihydroxybenzoyl)-β-D-glucopyranoside

* Unidentified; ^1^ likely, feruloyl hexose or hexoside of ferulic acid; ^2^ likely, sinapoyl hexose or hexoside of ferulic acid; ** doubly charged ion.

**Table 4:** Major compounds in the F3 and F4 and their tentative identification by HPLC–ESI–HRMS.

No.	t_R_, min	Formula	*m*/*z*	Δ*m*/*z*, ppm	Mode	Peak Area, ×10^7^		Assumed Compound
						F3	F4	
1	9.9	C_14_H_6_O_8_	300.9988	−0.70	ESI(−)	26.5	-	Ellagic acid
2	10.1	C_21_H_20_O_12_	463.0882	−0.07	ESI(−)	3.88	-	Isoquercetin
3	10.2	C_21_H_18_O_13_	477.0675	0.02	ESI(−)	5.03	-	Miquelianin
4	10.8	C_27_H_28_O_16_	607.1308	0.56	ESI(−)	1.19	-	Quercetin 3-[6″-(3-hydroxy-3-methylglutaryl) galactoside]
5	10.8	C_22_H_39_O_10_	463.2548	−0.24	ESI(−)	19.2	-	Isoliquiritin
6	11.0	C_21_H_18_O_12_	461.0726	0.05	ESI(−)	2.58	-	Kaempferol 3-glucuronide
7	11.5	C_21_H_16_O_13_	475.0516	−0.44	ESI(−)	13.9	-	Ellagic acid acetyl-xyloside
8	14.0	C_30_H_48_O_7_	519.3329	0.31	ESI(−)	2.18	-	Trachelosperogenin B
9	14.2	C_30_H_25_O_13_	593.1298	−0.41	ESI(−)	8.18	-	Tiliroside
10	14.8	C_30_H_48_O_7_	519.3330	0.54	ESI(−)	1.93	-	Platycodigenin
11	15.9 (2.9) *	C_30_H_48_O_6_	503.3376	−0.39	ESI(−)	15.4	0.02	19α-hydroxyasiatic acid
12	16.4 (3.3) *	C_30_H_48_O_6_	503.3376	−0.51	ESI(−)	18.8	0.02	Tomentosic acid
13	16.7	C_30_H_48_O_6_	503.3375	−0.69	ESI(−)	9.88	-	Myrianthic acid
14	17.3	C_30_H_48_O_6_	503.3377	−0.21	ESI(−)	5.56	-	1β-Hydroxyeuscaphic acid
15	17.5	C_30_H_46_O_6_	501.3222	0.08	ESI(−)	4.12	-	Triterpenoic acid with keto-group (isomer I)
16	18.0	C_30_H_46_O_6_	501.3221	−0.04	ESI(−)	5.32	-	Triterpenoic acid with keto-group (isomer II)
17	19.0 (5.3) *	C_30_H_48_O_5_	489.3571	−0.81	ESI(+)	26.4	0.04	Tormentic acid
18	19.3	C_33_H_56_O_14_	694.4007 [M + NH_4_]^+^	−0.20	ESI(+)	0.97	-	DGMG (18:3)
19	20.4	C_27_H_46_O_9_	532.3483 [M + NH_4_]^+^	0.60	ESI(+)	0.79	-	MGMG (18:3), isomer I
20	20.7	C_27_H_46_O_9_	532.3480 [M + NH_4_]^+^	0.03	ESI(+)	0.88	-	MGMG (18:3), isomer II
21	21.9	C_30_H_48_O_4_	473.3625	−0.05	ESI(+)	2.87	-	Corosolic acid
22	22.1	C_40_H_54_O_4_	599.4094	−0.10	ESI(+)	1.96	-	Erythroxanthin
23	23.4	C_40_H_54_O_4_	599.4097	0.30	ESI(+)	1.46	-	Halocynthiaxanthin
24	24.8 (11.8) *	C_19_H_38_O_4_	331.2842	−0.14	ESI(+)	2.10	2.40	Monopalmitin
25	25.2	C_39_H_64_O_11_	706.4523 [M + NH_4_]^+^	0.19	ESI(+)	1.35	-	MGDG (18:3/Oxo 12:0)
26	26.5 (13.5) *	C_21_H_42_O_4_	359.3156	0.16	ESI(+)	1.21	1.44	Monostearin
27	27.6 (14.4) *	C_51_H_84_O_15_	954.6149 [M + NH_4_]^+^	0.02	ESI(+)	49.8	0.09	DGDG (18:3/18:3)
28	28.1 (14.9) *	C_40_H_75_O_9_N	714.5512	−0.35	ESI(+)	18.8	0.04	Soyacerebroside I
29	28.3 (15.1) *	C_51_H_86_O_15_	956.6306 [M + NH_4_]^+^	0.06	ESI(+)	5.83	0.01	DGDG (18:3/18:2)
30	29.0 (15.8) *	C_49_H_86_O_15_	932.6307 [M + NH_4_]^+^	0.26	ESI(+)	23.5	0.09	DGDG (16:0/18:3)
31	29.1 (15.8) *	C_35_H_36_O_5_N_4_	593.2755	−0.67	ESI(+)	78.0	2.16	Pheophorbide a
32	29.8 (16.4) *	C_45_H_74_O_10_	792.5620 [M + NH_4_]^+^	−0.02	ESI(+)	98.6	0.28	MGDG (18:3/18:3)
33	30.4 (16.6) *	C_40_H_55_O	551.4249	0.33	ESI(+)	14.9	1.58	Anhydrolutein
34	30.5 (16.7) *	C_40_H_56_O_2_	568.4271	−0.63	ESI(+)	52.3	4.74	Lutein
35	30.5 (17.1) *	C_51_H_90_O_15_	960.6621 [M + NH_4_]^+^	0.32	ESI(+)	4.02	0.02	DGDG(18:3/18:0)
36	30.6 (17.2) *	C_45_H_76_O_10_	794.5774 [M + NH_4_]^+^	−0.35	ESI(+)	15.1	0.05	MGDG (18:3/18:2)
37	31.0 (17.6) *	C_43_H_73_O_11_	765.5151	−0.99	ESI(−)	28.2	0.06	1-(9Z,12Z,15Z-octadecatrienoyl)-2-hexadecanoyl-3-O-α-D-glucuronosyl-sn-glycerol (GlcADG(18:3/16:0)
38	31.3 (17.9) *	C_43_H_76_O_10_	770.5778 [M + NH_4_]^+^	0.11	ESI(+)	6.67	0.03	MGDG (16:0/18:3)
39	31.5 (18.1)*	C_35_H_78_O_10_	796.5934 [M + NH_4_]^+^	−0.26	ESI(+)	5.37	0.02	MDDG (18:3/18:0)
40	31.6 (18.1) *	C_36_H_38_O_5_N_4_	607.2913	−0.39	ESI(+)	21.4	13.7	Methyl pheophorbide a
41	32.4 (18.8) *	C_28_H_48_O_3_	433.3677	0.19	ESI(+)	2.55	0.03	2-(4-Hydroxyphenyl)ethyl icosanoate
42	32.4 (18.9) *	C_45_H_78_O_11_	793.5471	−0.02	ESI(−)	3.70	0.01	GlcADG(18:3/18:0)
43	33.4 (19.3) *	C_29_H_50_O_3_	447.3831	−0.41	ESI(+)	17.3	0.28	3,5-dihydroxystigmastan-6-one
44	33.4 (19.3) *	C_29_H_48_O_3_	445.3676	0.05	ESI(+)	12.2	3.33	5-Hydroxystigmastane-3,6-dione
45	34.0 (20.6) *	C_40_H_71_O_10_P	741.4711	−0.19	ESI(−)	20.2	0.23	1-(9Z,12Z,15Z-octadecatrienoyl)-2-(9Z-hexadecenoyl)-glycero-3-phospho-(1′-sn-glycerol) (PG(18:3/16:1))
46	36.7 (23.7) *	C_55_H_76_O_6_N_4_	887.5685	0.48	ESI(+)	-	29.3	Hydroxypheophytin A
47	38.5 (24.4) *	C_55_H_72_O_6_N_4_	885.5520	−0.49	ESI(+)	0.51	239	Pheophytin b
48	39.5 (25.2) *	C_55_H_74_O_5_N_4_	871.5726	−0.72	ESI(+)	0.33	2498	Pheophytin a

* Retention time in the analysis of the fraction F4.

## Data Availability

The data presented in this study are available in the article and Supplementary Materials.

## Supplementary Materials

The following supporting information can be downloaded at: https://www.mdpi.com/article/10.3390/metabo13050598/s1, Figure S1: A cloudberry (*Rubus chamaemorus* L.) plant at the collection site; Figure S2: HPLC–HRMS chromatograms (total ion current) of the cloudberry leaf extract fractions F2–F4; Table S1: Annotation of the major cross-peaks in HSQC NMR spectra of the cloudberry leaf extract fractions F2–F4.

## References

[1] Thiem B. (2003). *Rubus chamaemorus* L.—A boreal plant rich in biologically active metabolites: A review. Biol. Lett..

[2] Whaley A.K., Ponkratova A.O., Teslov L.S., Luzhanin V.G. (2020). Review of cloudberry secondary metabolites and their biological activity. Med. Pharm. J..

[3] Kähkönen M., Kylli P., Ollilainen V., Salminen J., Heinonen M. (2012). Antioxidant Activity of Isolated Ellagitannins from Red Raspberries and Cloudberries. J. Agric. Food Chem..

[4] Määttä-Riihinen K.R., Kamal-Eldin A., Törrönen A.R. (2004). Identification and Quantification of Phenolic Compounds in Berries of Fragaria and Rubus Species (Family Rosaceae). J. Agric. Food Chem..

[5] Ferlemi A.-V., Lamari F.N. (2016). Berry Leaves: An Alternative Source of Bioactive Natural Products of Nutritional and Medicinal Value. Antioxidants.

[6] Mertz C., Cheynier V., Günata Z., Brat P. (2007). Analysis of phenolic compounds in two blackberry species (*Rubus glaucus* and *Rubus adenotrichus*) by high-performance liquid chromatography with diode array detection and electrospray ion trap mass spectrometry. J. Agric. Food Chem..

[7] Renai L., Scordo C.V.A., Chiuminatto U., Ulaszewska M., Giordani E., Petrucci W.A., Tozzi F., Nin S., Del Bubba M. (2021). Liquid Chromatographic Quadrupole Time-of-Flight Mass Spectrometric Untargeted Profiling of (Poly)phenolic Compounds in *Rubus idaeus* L. and *Rubus occidentalis* L. Fruits and Their Comparative Evaluation. Antioxidants.

[8] Kähkönen M.P., Hopia A.I., Heinonen M. (2001). Berry phenolics and their antioxidant activity. J. Agric. Food Chem..

[9] Afrin S., Giampieri F., Gasparrini M., Forbes-Hernandez T.Y., Varela-López A., Quiles J.L., Mezzetti B., Battino M. (2016). Chemopreventive and Therapeutic Effects of Edible Berries: A Focus on Colon Cancer Prevention and Treatment. Molecules.

[10] Puupponen-Pimiä R., Nohynek L., Suvanto J., Salminen J.-P., Seppänen-Laakso T., Tähtiharju J., Honkapää K., Oksman-Caldentey K.-M. (2021). Natural Antimicrobials from Cloudberry (*Rubus chamaemorus*) Seeds by Sanding and Hydrothermal Extraction. ACS Food Sci. Technol..

[11] Rocabado G.O., Bedoya L.M., Abad M.J., Bermejo P. (2008). Rubus—A review of its phytochemical and pharmacological profile. Nat. Prod. Commun..

[12] Lashmanova E.A., Kuzivanova O.A., Dymova O.V., Moskalev A.A. (2019). The Effects of Cloudberry Fruit Extract on Drosophila Melanogaster Lifespan and Stress Resistance. Adv. Gerontol..

[13] McDougall G.J., Martinussen I., Junttila O., Verrall S., Stewart D. (2011). Assessing the influence of genotype and temperature on polyphenol composition in cloudberry (*Rubus chamaemorus* L.) using a novel mass spectrometric method. J. Agric. Food Chem..

[14] Thiem B., Goslinska J. (2004). Antimicrobial activity of *Rubus chamaemorus* leaves. Fitoterapia.

[15] Whaley A.K., Ponkratova A.O., Orlova A.A., Serebryakov E.B., Smirnov S.N., Proksh P., Ionov N.S., Poroikov V.V., Luzhanin V.G. (2021). Phytochemical Analysis of Polyphenol Secondary Metabolites in Cloudberry (*Rubus chamaemorus* L.) Leaves. Pharm. Chem. J..

[16] Faleva A.V., Ul’yanovskii N.V., Falev D.I., Onuchina A.A., Budaev N.A., Kosyakov D.S. (2022). New Oligomeric Dihydrochalcones in the Moss Polytrichum commune: Identification, Isolation, and Antioxidant Activity. Metabolites.

[17] Ul’yanovskii N.V., Onuchina A.A., Faleva A.V., Gorbova N.S., Kosyakov D.S. (2022). Comprehensive Characterization of Chemical Composition and Antioxidant Activity of Lignan-Rich Coniferous Knotwood Extractives. Antioxidants.

[18] Faleva A.V., Pikovskoi I.I., Pokryshkin S.A., Chukhchin D.G., Kosyakov D.S. (2022). Features of the Chemical Composition and Structure of Birch Phloem Dioxane Lignin: A Comprehensive Study. Polymers.

[19] Jerković I., Cikoš A.-M., Babić S., Čižmek L., Bojanić K., Aladić K., Ul’yanovskii N.V., Kosyakov D.S., Lebedev A.T., Čož-Rakovac R. (2021). Bioprospecting of Less-Polar Constituents from Endemic Brown Macroalga *Fucus virsoides* J. Agardh from the Adriatic Sea and Targeted Antioxidant Effects In Vitro and In Vivo (Zebrafish Model). Mar. Drugs.

[20] Ul’yanovskii N.V., Falev D.I., Kosyakov D.S. (2022). Highly sensitive ligand exchange chromatographic determination of apiose in plant biomass. Microchem. J..

[21] Singleton V.L., Rossi J.A. (1965). Colorimetry of total phenolics with phosphomolybdic-phosphotungstic acid reagents. Am. J. Enol. Vitic..

[22] Yashin A.Y. (2008). A flow-injection system with amperometric detection for selective determination of antioxidants in food-stuffs and drinks. Russ. J. Gen. Chem..

[23] Pegg R.B., Amarowicz R., Naczk M., Shahidi F. (2007). PHOTOCHEM^®^ for determination of antioxidant capacity of plant extracts. ACS Symp. Ser..

[24] Ghitescu R.-E., Volf I., Carausu C., Bühlmann A.-M., Gilca I.A., Popa V.I. (2015). Optimization of ultrasound-assisted extraction of polyphenols from spruce wood bark. Ultrason. Sonochem..

[25] Vongsak B., Sithisarn P., Mangmool S., Thongpraditchote S., Wongkrajang Y., Gritsanapan W. (2013). Maximizing total phenolics, total flavonoids contents and antioxidant activity of Moringa oleifera leaf extract by the appropriate extraction method. Ind Crops Prod..

[26] Ha Y.W., Lim S.S., Ha I.J., Na Y.-C., Seo J.-J., Shin H., Son S.H., Kim Y.S. (2007). Preparative isolation of four ginsenosides from Korean red ginseng (steam-treated Panax ginseng C. A. Meyer), by high-speed counter-current chromatography coupled with evaporative light scattering detection. J. Chromatogr. A.

[27] Tzanova M., Atanasov V., Yaneva Z., Ivanova D., Dinev T. (2020). Selectivity of current extraction techniques for flavonoids from plant materials. Processes.

[28] Baardseth P., Russwurm H. (1978). Content of some organic acids in cloudberry (*Rubus chamaemorus* L.). Food Chem..

[29] Koponen J.M., Happonen A.M., Mattila P.H., Törrönen A.R. (2007). Contents of Anthocyanins and Ellagitannins in Selected Foods Consumed in Finland. J. Agric. Food Chem..

[30] Martić A., Čižmek L., Ul’yanovskii N.V., Paradžik T., Perković L., Matijević G., Vujović T., Baković M., Babić S., Kosyakov D.S. (2023). Intra-Species Variations of Bioactive Compounds of Two Dictyota Species from the Adriatic Sea: Antioxidant, Antimicrobial, Dermatological, Dietary, and Neuroprotective Potential. Antioxidants.

[31] Yuan G.-F., Chen X.-E., Li D. (2014). Conjugated linolenic acids and their bioactivities: A review. Food Funct..

