# Supplementary material for: Comprehensive Characterization of Secondary Metabolites in Fruits and Leaves of Cloudberry (Rubus chamaemorus L.)
Source: Metabolites. 2023 Apr 27;13(5):598. doi: 10.3390/metabo13050598 (PMC10222136; doi:10.3390/metabo13050598)
Supplement: Supplementary file 1 [file metabolites-13-00598-s001.zip › metabolites-2361354-supplementary.pdf]

Supplementary material

Comprehensive Characterization of Secondary Metabolites in Fruits and Leaves of Cloudberry (*Rubus chamaemorus L.*)

Anna V. Faleva, Nikolay V. Ul'yanovskii*, Aleksandra A. Onuchina, Danil I. Falev and Dmitry S. Kosyakov*

Laboratory of Natural Compounds Chemistry and Bioanalytics, Core Facility Center "Arktika", M.V. Lomonosov Northern (Arctic) Federal University, Northern Dvina Emb. 17, 163002 Arkhangelsk, Russia;

*Correspondence: n.ulyanovsky@narfu.ru (N.V.U.); d.kosyakov@narfu.ru (D.S.K.)

Contents:

Figure S1. A cloudberry (*Rubus chamaemorus* L.) plant at the collection site.

Figure S2. HPLC-HRMS chromatograms (total ion current) of the cloudberry leaf extract fractions F2-F4.

Table S1. Annotation of the major cross-peaks in HSQC NMR spectra of the cloudberry leaf extract fractions F2-F4.

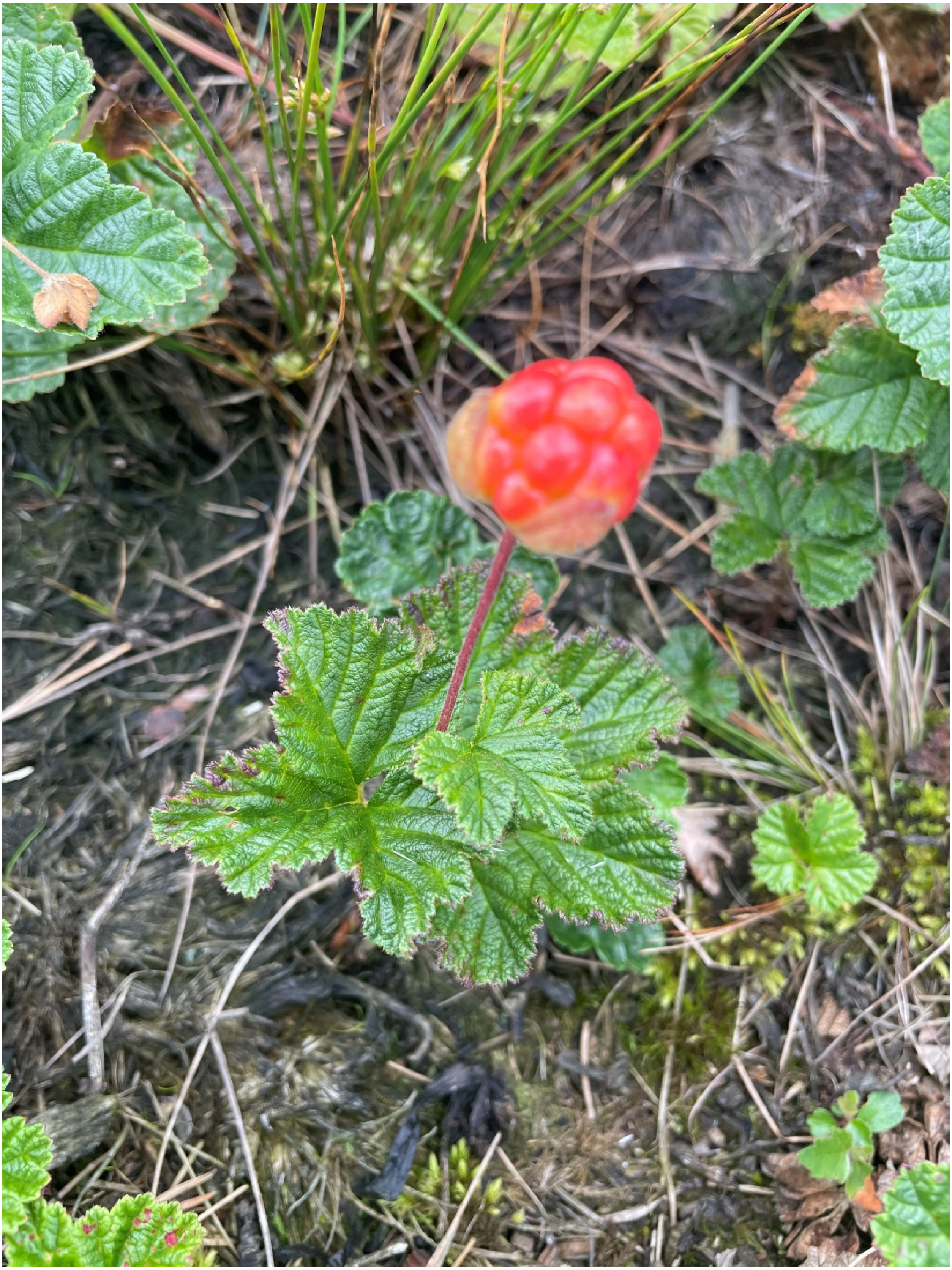

Figure S1. A cloudberry (*Rubus chamaemorus* L.) plant at the collection site.

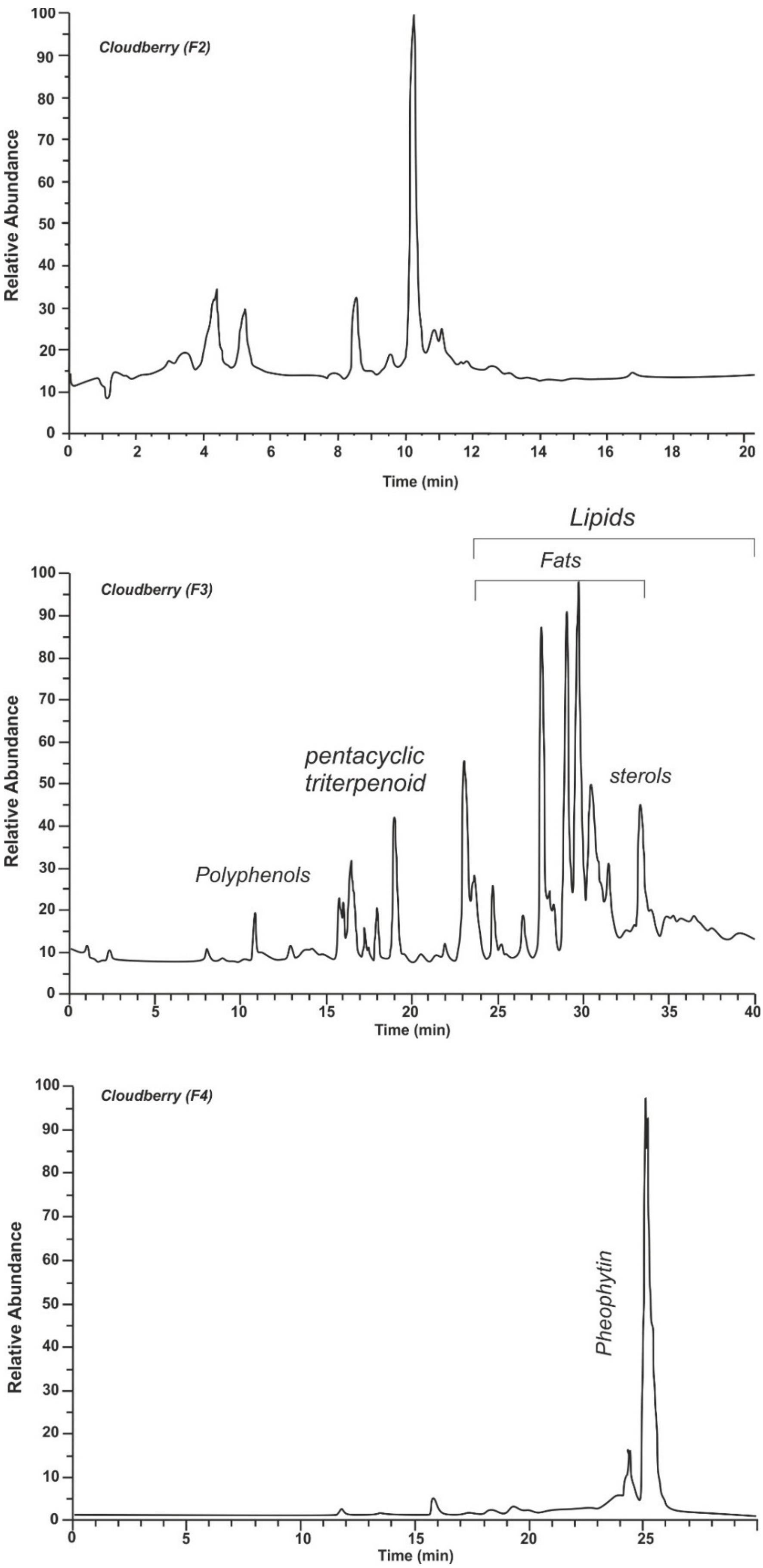

Figure S2. HPLC-HRMS chromatograms (total ion current) of the cloudberry leaf extract fractions F2-F4.

Table S1. Annotation of the major cross-peaks in HSQC NMR spectra of the cloudberry leaf extract fractions F2-F4.

**Name of compounds**	**Characteristic NMR peaks, ppm (δC/δH)**
F2 (DMSO-d_6)	
Miquelianin	116.50/7.81; 121.13/7.52; 93.41/6.41; 98.58/6.21; 101.35/5.43
Procyanidin/Catechin	115.17/6.84; 118.10/6.65; 94.97/5.89; 95.58/5.67; 77.95/4.73; 64.94/4.05; 28.96/2.75-2.53
Carbohydrates	92.37/4.91; 96.77/4.27; 75.18/3.51; 71.49/3.33; 73.76/3.28; 76.31/3.28; 70.13/3.04; 60.89/3.66-3.44
Ellagitannins	109.05/7.05
Cinnamic acid:	
- Caffeic acid	114.84/7.05; 121.35/7.01; 113.77/6.26; 145.20/7.47
- Ferulic acid	110.61/7.33; 123.10/7.11; 145.36/7.55; 114.59/6.39
- Sinapic acid	106.16/7.03; 145.36/7.55; 114.59/6.39
- *p*-coumaric acid	127.91/7.35; 145.36/7.55; 114.59/6.39
OMe-groups	55.49/3.74
F3 (DMSO-d_6)	
Procyanidin	114.63-115.36/6.84-6.78
Carbohydrates	99.12/4.68; 103.47/4.10; 77.54/3.16; 73.07/3.28; 72.75/3.56; 60.12/3.51-3.46
Triacylglyceride (unsaturated fatty acids)	69.67/5.10; 61.97/4.31-4.12; (127.53/5.31; 25.04/2.76; 33.24/2.26; 24.15/1.49; 28.67/1.23; 21.78/1.25; 13.63/0.84)
Pentacyclic triterpenic acids	126.73/5.18; 53.40/2.37; 24.96/2.49; 46.66/1.66; 40.97/1.40-1.13; 54.88/0.76; 26.23/1.08; 16.96/0.67
Carotenoids	137.21/6.38; 132.67/6.32; 145.82/5.84; 130.40/6.16; 11.63/1.97
OMe groups	55.41/3.74
F4 (CDCl-d_1)	
Acylglycerides (unsaturated fatty acids)	68.16/5.28; 61.37/4.31-4.16; (127.44/5.37; 24.91/2.82; 33.68/2.31; 24.26/1.62; 28.49/1.32; 21.99/1.30; 13.66/0.89)
Sitosterol-like compounds	11.10/0.69; 11.19/0.86; 41.46/2.30-2.24; 45.19/0.94; 49.37/0.95; 55.23/1.11; 56.07/1.01; 70.93/3.52 121.3/5.37;
Pheophytin	10.95/3.34; 12.04/3.69; 64.06/6.28;93.34/8.81; 99.88/9.78; 106.40/10.01; 124.9/6.27; 127.47/7.87
